# Risk-Adapted Lung Cancer Screening Starting Ages for Former Smokers

**DOI:** 10.1001/jamanetworkopen.2025.51281

**Published:** 2025-12-23

**Authors:** Clara Frick, Lára R. Hallsson, Uwe Siebert, Megha Bhardwaj, Ben Schöttker, Hermann Brenner

**Affiliations:** 1 Division of Clinical Epidemiology of Early Cancer Detection, German Cancer Research Center (DKFZ), Heidelberg, Germany; 2 Heidelberg Medical Faculty, Heidelberg University, Heidelberg, Germany; 3 Institute of Public Health, Medical Decision Making, and Health Technology Assessment, Department of Public Health, Health Services Research, and Health Technology Assessment, UMIT Tirol–University for Health Sciences and Technology, Tirol, Austria; 4 Center for Health Decision Science, Harvard T. H. Chan School of Public Health, Boston, Massachusetts; 5 Institute for Technology Assessment, Massachusetts General Hospital, Harvard Medical School, Boston, Massachusetts; 6 Department of Epidemiology, Harvard T. H. Chan School of Public Health, Boston, Massachusetts; 7 Department of Health Policy and Management, Harvard T. H. Chan School of Public Health, Boston, Massachusetts; 8 Department of Radiology, Massachusetts General Hospital, Harvard Medical School, Boston, Massachusetts; 9 Cancer Prevention Graduate School, German Cancer Research Center (DKFZ), Heidelberg, Germany

## Abstract

**Importance:**

The US Preventive Services Task Force (USPSTF) recommends lung cancer screening for individuals aged 50 to 80 years with a smoking history of at least 20 pack-years and 15 quit-years or less. This recommendation implies that former heavy smokers with at least 20 pack-years would be offered screening from 50 years of age onward or not at all, depending on a dichotomous classification by time since smoking cessation.

**Objective:**

To develop a more personalized lung cancer screening strategy that reflects individual risks by defining risk-adapted starting ages for screening according to time since cessation.

**Design, Setting, and Participants:**

This cohort study used longitudinal data from the population-based UK Biobank, which recruited participants in England, Wales, and Scotland between 2006 and 2010. Participants were 50 years or older, had no prior cancer diagnosis, and had at least 20 pack-years of smoking history. The following censoring dates were applied: December 31, 2020, for England; November 30, 2021, for Scotland; and December 31, 2016, for Wales. The present analysis was performed in April 2025.

**Exposure:**

Duration of smoking cessation (categorized into ≤5, 6-10, 11-15, and >15 quit-years).

**Main Outcomes and Measures:**

The association between smoking cessation time, pack-years, age, and 10-year lung cancer incidence was assessed using multivariable extended Cox proportional hazards models. Risk postponement periods (RPPs) were estimated from the regression coefficients for time since cessation and age. These RPP estimates were used to derive risk-adapted starting ages for lung cancer screening among former heavy smokers, using the USPSTF-proposed starting age of 50 years as a reference for the risk level of continuing heavy smokers.

**Results:**

A total of 86 035 former or current heavy smokers (mean [SD] age, 60.8 [5.3] years; range, 50-72 years), of whom 2109 (2.5%) were diagnosed with lung cancer, were included in the analysis. The RPPs for smoking cessation were 2.7 (95% CI, 0.0-5.3), 6.2 (95% CI, 4.4-7.9), 10.4 (95% CI, 8.5-12.4), and 17.1 (95% CI, 15.0-19.2) years for former heavy smokers who quit at least 5, 6 to 10, 11 to 15, and more than 15 years ago, respectively. This could translate to risk-adapted screening starting ages ranging from 53 (95% CI, 50-55) years to 67 (95% CI, 65-69) years, if current smokers begin screening at age 50.

**Conclusions and Relevance:**

This cohort study of the UK Biobank provides an empirical basis for risk-adapted starting ages for lung cancer screening among former heavy smokers.

## Key Points

**Question:**

Considering their risk compared with current smokers, when should former smokers begin lung cancer screening?

**Findings:**

In this cohort study of 86 035 former or current heavy smokers, former heavy smokers could be screened between 3 and 17 years later than current heavy smokers, depending on the length of smoking cessation, in order to more closely reflect their risk levels. Whereas the US Preventive Services Task Force currently recommends a unified starting age of 50 years, the derived risk-adapted screening start ages for former heavy smokers ranged between 53 and 67 years of age.

**Meaning:**

This study’s findings suggest that using differentiated, risk-adapted starting ages of former smokers could allow for enhanced lung cancer screening strategies.

## Teaser

This cohort study seeks to define risk-adapted starting ages for personalized lung cancer screening by examining associations between smoking cessation time, pack-years, age, and 10-year lung cancer incidence.

## Introduction

Lung cancer is the leading cause of cancer-related mortality globally, accounting for 1.8 million deaths annually.^1^ Randomized trials have demonstrated that screening with low-dose computed tomography (LDCT) can reduce lung cancer mortality by 20% to 30%.^2,3^ LDCT screening programs are therefore being piloted in an increasing number of countries.^4^ However, LDCT screening not only offers benefits but also carries potential harms, as it can lead to false-positive findings at screening, overdiagnosis, and overtreatment.^5^ Hence, targeting screening to those at highest risk is crucial to maximize the benefit-harm balance of lung cancer screening.

The US Preventive Services Task Force (USPSTF) recommends annual LDCT screening for high-risk individuals, defined as ever smokers aged 50 to 80 years who have a lifetime smoking exposure of at least 20 pack-years and, in the case of former smokers, who have quit within the past 15 years.^6^ This recommendation implies a common starting age of lung cancer screening at 50 years for heavy smokers (≥20 pack-years) who either still smoke at age 50 years or quit within the last 15 years, and no screening offered to former heavy smokers (≥20 pack years) who quit more than 15 years ago. However, this simple dichotomy for former heavy smokers fails to account for the gradual decline in lung cancer risk with increasing time since cessation and the counteracting increase in lung cancer risk with age, despite continued smoking abstinence.^7^ On one hand, former heavy smokers who quit less than 15 years ago may already have a substantially reduced lung cancer risk compared with continuing heavy smokers and would be expected to reach a comparable lung cancer risk at an older age than 50 years. On the other hand, due to the increase in lung cancer risk with age, former heavy smokers who quit more than 15 years ago may at some age still reach the same lung cancer risk as current heavy smokers have at age 50, and could therefore still benefit from lung cancer screening from that age onwards.

The aim of this study was to estimate when former heavy smokers (≥20 pack years) could initiate lung cancer screening based on their risk compared with current smokers and derive risk-adapted starting ages based on time since smoking cessation.

## Methods

### Study Design and Study Population

Our analysis is based on longitudinal data from the UK Biobank (UKB). In this prospective cohort study from the UK (England, Scotland, and Wales), approximately 500 000 participants between 40 and 72 years of age at baseline (with a few outliers in the range of 37-73 years) were recruited in the years 2006 to 2010.^8^ Baseline data collection included a detailed self-reported lifetime history of smoking.^9^ Health-related outcomes are regularly updated by using electronic health records from the UK National Health Service, which includes information on cancer, primary care, hospital admissions, and death. We restricted our analysis to participants aged 50 years or older, with no prior cancer diagnosis, and with a cumulative smoking exposure of 20 or more pack-years at enrollment, to more closely reflect the target population of general screening programs. Cancer diagnoses were defined by the following *International Statistical Classification of Diseases and Related Health Problems, Tenth Revision* (*ICD-10*) codes (or equivalent *International Classification of Diseases, Ninth Revision* codes): C00-14, C15-26, C30-39, C40-41, C43, C45-49, C50, C51-58, C60-63, C64-68, C69-72, C73-75, C76-80, C81-96, and C97. Prevalent nonmelanoma skin cancers and benign neoplasms were not excluded.

The UKB obtained ethical approval from the North-West Multicenter Research Ethics Committee. The authors obtained ethical approval from the ethics committee of the Heidelberg Medical Faculty of Heidelberg University for this analysis. Written informed consent was obtained from all study participants. The reporting of this study was conducted in accordance with the Strengthening the Reporting of Observational Studies in Epidemiology (STROBE) reporting guideline.

### Study Variables and Outcome

For this study, participants were classified according to smoking history as current heavy smokers or as former heavy smokers who quit within the last 5 years, 6 to 10 years ago, 11 to 15 years ago, or more than 15 years ago.^8^ The study outcome was first diagnosis of lung cancer during the follow-up period, identified using the *ICD-10* code of C34. Follow-up for cancer outcomes varied by regional cancer registries. The following censoring dates were applied: December 31, 2020, for England; November 30, 2021, for Scotland; and December 31, 2016, for Wales.

### Statistical Analysis

The statistical analysis was conducted in April 2025. Following the approach of a previous UKB analysis,^10^ missing values for age at smoking initiation were replaced by 17 years, the mode around which the known starting age values were tightly clustered. Missing smoking values (cigarettes per day and years since smoking cessation) were then imputed using predictive mean matching. To prevent imputed quit-years from exceeding the current age, we instead imputed the proportion of time since starting smoking that corresponded to cessation time. Quit-years and smoking cessation ages were derived from the imputed proportion. Additional variables included in the imputation model were sex, age, body mass index, education, ethnicity, smoking status, and age at smoking initiation. Results from 5 imputed datasets were pooled using Rubin rules.^11^

Pack-years were calculated by multiplying the average number of cigarette packs smoked per day (assuming a package is composed of 20 cigarettes) with the total number of years the individual has smoked. For current smokers, years smoked were calculated as the difference between current age and age at smoking initiation. For former smokers, years smoked were calculated as the difference between age at cessation and age at initiation. Given the available data, quit-years were defined as the difference between current age and reported age at smoking cessation, representing the years quit at baseline (rather than a cumulative total over multiple quit attempts). For individuals who quit within the same year as recruitment, we approximated time since cessation as half a year.

Descriptive statistics were used to characterize the study population of ever heavy smokers according to the distribution of smoking exposure, sex, and age. Extended Cox proportional hazards models were used to assess the association between age (continuous variable) and time since smoking cessation (categories: continuing smoker [reference group] or quit ≤5, 6-10, 11-15, or more than 15 years ago) and time to lung cancer. Time since baseline (up to 10 years) was used as the time scale. Models were adjusted for sex and number of pack-years reported at baseline (continuous). Given that time since smoking cessation increases during follow-up among former smokers, it was entered as a time-dependent exposure variable in the Cox models. This was implemented by incorporating the baseline quit-year values dynamically, assuming that they increased by one unit for each additional year of follow-up.

For a small subset of the study population living in an area surrounding UKB’s coordinating center in Stockport, updated information on smoking status obtained during follow-up was available, which was considered in the analysis by including smoking status as a time-dependent covariate in the Cox models. In cases of inconsistent responses, baseline smoking status was prioritized. For example, participants who reported a smoking history at baseline but later indicated never having smoked were classified as having quit during follow-up.

We used the concept of risk postponement periods (RPPs) to determine the age at which former heavy smokers would reach the same lung cancer risk as continuing heavy smokers. The concept can be considered as a variant (for protective factors, such as smoking cessation, rather than risk factors) of the long-established concept of risk advancement periods (RAPs), which is applicable to chronic diseases whose risk monotonically increases with age (as is the case for lung cancer) and has been described in detail elsewhere.^12^ Briefly, like RAPs, RPPs can be easily derived from multivariable regression models as the ratio of the absolute value of the regression coefficient for the protective factor under investigation and for age (entered in the regression model in years). In our context, RPPs were derived for the various categories of years since smoking cessation as the ratios of the absolute values of the regression coefficients for these categories and the regression coefficient for age. Risk-adapted starting ages for lung cancer screening were then obtained by adding these RPPs to the reference starting age of 50 years that has been proposed by the USPSTF. We calculated 95% CIs for the RPPs and the risk-adapted starting ages as previously described.^12^

In the Cox model, the proportional hazards assumption was assessed through inspection of Schoenfeld residuals plots to evaluate whether analyses would need to be stratified by time. To assess the impact of excluding individuals younger than 50 years and with less than 20 pack-years of smoking history, we conducted a sensitivity analysis that included participants of all ages and pack-years of smoking. Statistical tests were 2-sided with an α of .05. All analyses and visualizations were conducted using the statistical software R, version 4.3.3 (R Foundation) and the R packages dplyr^13^ and mice.^14^

## Results

### Characteristics of Study Population

A total of 86 035 former or current heavy smokers (≥20 pack-years) were included in the analysis (Figure), of whom 2109 (2.5%) developed lung cancer during a maximum of 10 years of enrollment (Table 1). Among the included participants, there were more males (51 298 [59.6%]) than females (34 737 [40.4%]). Participants had a mean (SD) age of 60.8 (5.3) years (range, 50-72 years). The age group of 60 to 69 years made up the largest proportion of the cohort, including 53 365 participants (62.1%) and 1581 (75.0%) of the total lung cancer cases. Current smokers and individuals with more than 15 quit-years each constituted about one-third of the study population, with the remainder of the different smoking cessation time categories constituting between 10% and 20% of the participants. A total of 1836 lung cancer cases (87.0%) occurred among individuals eligible for screening according to USPSTF criteria, with 243 cases (12.9%) found in the group of individuals who quit smoking more than 15 years ago. Information on missing data is available in (eTable 1 in Supplement 1).

**Figure.**
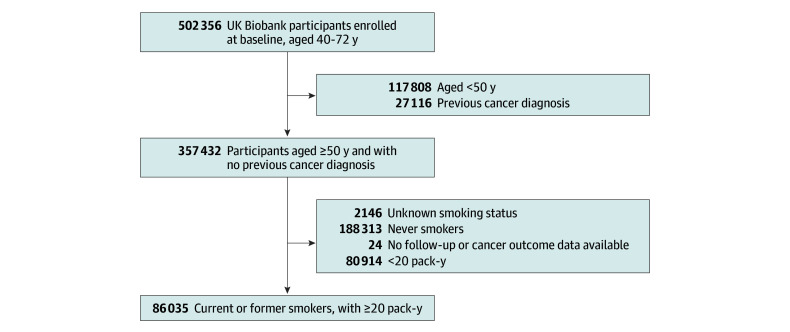
Selection of Study Participants From the UK Biobank Cohort

**Table 1.** Baseline Characteristics of Study Participants After Imputation of Missing Smoking Data

Characteristic^a^	Study participants, No. (%)	
	Total (N = 86 035)	Lung cancer cases (n = 2109)
Sex		
Female	34 737 (40.4)	843 (40.0)
Male	51 298 (59.6)	1266 (60.0)
Age at baseline, y		
50-59	32 068 (37.3)	512 (24.3)
60-69	53 365 (62.1)	1581 (75.0)
≥70	602 (0.7)	16 (0.8)
Smoking status at baseline^b^		
Current	25 215 (29.3)	1060 (50.3)
Former, ≤5 quit-y	16 256 (18.9)	385 (18.3)
Former, 6-10 quit-y	11 801 (13.7)	252 (12.0)
Former, 11-15 quit-y	9488 (11.0)	139 (6.6)
Former, >15 quit-y	23 275 (27.1)	273 (12.9)
Years smoked at baseline^b^		
<15	1882 (2.2)	12 (0.6)
15-24	14 289 (16.6)	111 (5.3)
25-34	24 088 (28.0)	308 (14.6)
35-44	31 180 (36.3)	864 (41.0)
≥45	14 596 (17.0)	814 (38.6)
Cigarettes smoked per d		
≤10	5000 (5.8)	169 (8.0)
11-20	51 570 (60.0)	1206 (57.2)
21-30	18 036 (21.0)	449 (21.3)
≥30	11 429 (13.3)	285 (13.5)
Pack-years^b^		
20-29	31 756 (36.9)	450 (21.3)
30-39	24 033 (27.9)	492 (23.3)
40-49	14 761 (17.2)	512 (24.3)
≥50	15 485 (18.0)	655 (31.1)
Eligible by USPSTF 2021 criteria^c^	62 760 (73.0)	1836 (87.0)

Abbreviation: USPSTF, US Preventive Services Task Force.

^a^ Characteristics of study participants presented are derived from the first imputed dataset.

^b^ Years are rounded to the nearest whole number.

^c^ Lung cancer screening eligibility by USPSTF 2021 criteria is as follows: 50 to 80 years of age, 20 or more pack-years, and no more than 15 quit-years.

Updated information on smoking status and age at smoking cessation was available for 6307 (7.3%) of the included study participants after a median follow-up time of 6.4 years (IQR, 4.7-8.6 years). Of the 1368 participants who were current smokers at baseline, 664 (48.5%) had quit, and of 4939 participants who were former smokers, 111 (2.2%) had resumed smoking.

### Lung Cancer Risk, RPPs, and Risk-Adapted Starting Ages of Screening for Former Heavy Smokers

Overall, 2109 incident lung cancer cases were observed; 1060 (50.3%) were among current smokers and 1049 (49.7%) among former smokers at baseline. Prolonged smoking cessation times were associated with a substantial risk reduction and delay of lung cancer (Table 2). No departures of the Cox proportional hazards assumption were observed in the Schoenfeld residuals plots. Hazard ratios (HRs) ranged from 0.80 (95% CI, 0.64-1.00) for individuals who quit smoking within the last 5 years to 0.25 (95% CI, 0.22-0.28) for those who quit more than 15 years ago. Among recent former smokers (quit-time of 5 years or less), the estimated RPP was 2.7 (95% CI, 0.0-5.3) years. Compared with the risk level of current smokers at age 50 years, these individuals reached the same level of risk and could be screened at age 53 (95% CI, 50-55) years. For individuals who quit 6 to 10 years ago, from the HR of 0.60 (95% CI, 0.52-0.69), an estimated RPP of 6.2 (95% CI, 4.4-7.9) years was obtained, from which the risk-adapted starting age of 56 (95% CI, 54-58) years was calculated. With a longer quit-time of 11 to 15 years, the HR of 0.42 (95% CI, 0.37-0.49) corresponded to an RPP of 10.4 (95% CI, 8.5-12.4) years. These individuals reached comparable risk levels and could therefore begin screening at age 60 (95% CI, 59-62) years. Among long-term former smokers (quit-time of at least 15 years), an HR of 0.25 (95% CI, 0.22-0.28) yielded an RPP of 17.1 (95% CI, 15.0-19.2) years, translating to a screening starting age at comparable risk level of 67 (95% CI, 65-69) years.

**Table 2.** Hazard Ratios (HRs), Risk Postponement Periods (RPPs), and Risk-Adapted Starting Ages of Screening Among Former Smokers, According to Years Since Quitting Smoking

Characteristic	Person-y	Lung cancer cases	HR (95% CI)^a^	RPP (95% CI), y	Risk-adapted starting age (95% CI), y
Age, y	NA	NA	1.09 (1.08-1.10)	NA	NA
Time since smoking cessation, y					
0	244 014	1053	1 [Reference]^b^	NA	50^c^
≤5	39 985	99	0.80 (0.64-1.00)	2.7 (0.0-5.3)	53 (50-55)^c^
6-10	104 367	270	0.60 (0.52-0.69)	6.2 (4.4-7.9)	56 (54-58)^c^
11-15	118 174	250	0.42 (0.37-0.49)	10.4 (8.5-12.4)	60 (59-62)^c^
>15	339 041	437	0.25 (0.22-0.28)	17.1 (15.0-19.2)	67 (65-69)^d^

Abbreviation: NA, not applicable.

^a^ The HRs were adjusted for pack-years of smoking history at baseline.

^b^ Participants who currently smoke with at least 20 pack-years of smoking exposure were used as a reference.

^c^ Indicates US Preventive Services Task Force (USPSTF) eligibility from 50 years of age onward.

^d^ Indicates ineligibility according to USPSTF screening recommendations.

Including participants younger than 50 and those with less than 20 pack-years of smoking history yielded consistent RPPs, ranging between 3.2 (95% CI, 1.2-5.2) years and 15.3 (95% CI, 14.1-16.4) years. These corresponded to starting ages of 53 (95% CI, 51-55) years for those with 5 or fewer quit-years, 56 (95% CI, 55-57) years for 6 to 10 quit-years, 59 (95% CI, 58-60) years for 11 to 15 quit-years, and 65 (95% CI, 64-66) years more than 15 quit-years (eTables 2 and 3 in Supplement 1).

## Discussion

Our cohort study provides an empirical basis for a more personalized, evidence-based approach to lung cancer screening for former heavy smokers, using RPPs derived from smoking cessation time to replace a unified starting age of 50 years with risk-adapted starting ages. A quit time of 5 years or less translated to an RPP of 2.7 (95% CI, 0.0-5.3) years, and more than 15 quit-years translated to an RPP of 17.1 (95% CI, 15.0-19.2) years. Whereas a starting age of 50 years has been implied by USPSTF eligibility criteria, our study proposes alternative starting ages for former heavy smokers, ranging from 53 to 67 years, depending on the length of time since smoking cessation. Notably, our findings suggest that based on quantifiable RPPs, long-term quitters of 15 years or longer with a lifetime exposure of 20 or more pack-years might still benefit from initiating screening at 67 (95% CI, 65-69) years of age.

The present analysis was based on taking 50 years as the starting point for lung cancer screening among current heavy smokers. In the 2021 update to the USPSTF screening eligibility criteria, the starting age for screening was lowered from 55 to 50 years.^6^ This change was largely informed by the Dutch-Belgian randomized clinical lung cancer screening trial (NELSON), the largest of its kind in Europe, which showed that computed tomography screening could substantially lower lung cancer mortality in the age group of 50 to 74 years.^3^ However, the NELSON trial, like many other lung cancer screening trials, did not include participants with more than 10 years since smoking cessation. Half of the study participants were former smokers, but most of them had quit recently, within 5 years (62%), whereas about 36% had quit within 6 to 10 years. Thus, the USPSTF starting age recommendation is sustained by evidence from a study population consisting mostly of current and recent smokers and including no long-term quitters. Our proposed starting ages for former smokers are contingent on the accuracy of 50 years of age reflecting the risks of current heavy smokers. Because the NELSON trial included a substantial number of recent former smokers,^3^ the assumption that 50 years is an ideal starting point for screening current smokers may not hold and could even be earlier, which would suggest that our proposed starting ages for former smokers might be slightly overestimated. Ultimately, our study demonstrates that starting ages should be tailored for former smokers and can be updated as new evidence emerges from trials on the optimal age for screening initiation among current smokers.

Several authors have raised concerns about excluding former heavy smokers who quit more than 15 years ago from screening, given that aging and a heavy smoking history slow the reduction of lung cancer risk from smoking cessation.^7,15,16^ The risk-adapted starting ages derived in our analysis may serve as a means to address these concerns. At the same time, our study also suggests risk-adapted postponement of starting ages for former heavy smokers who quit less than 15 years ago. Such a postponement has also been proposed in a recent analysis of a prospective cohort study from China.^17^ This study found that while men with at least 20 pack-years reached a 10-year cumulative risk of 1.37% by 50 years of age, male former smokers who quit within 15 years were found to attain this risk threshold at 52 years.^17^ However, no further differentiation by time since cessation was performed.

Risk-adapted variation of the initiation age for lung cancer screening, as suggested by our analysis, may help mitigate potential harms of population-wide lung cancer screening programs and maximize the benefits of widening eligibility criteria. Including high-risk long-term former smokers would help reduce the number of unscreened cases and late-stage diagnoses. Additionally, delaying screening until the individual’s risk of lung cancer substantially increases to yield an appropriate benefit-harm balance, rather than including them at the same age as heavy current smokers, may lower unnecessary psychological and physical burdens, such as false-positive findings, radiological exposure, overdiagnosis, and overtreatment, and simultaneously optimize cost-effectiveness. RPPs according to quit-time may additionally serve as a communication tool incentivizing the benefits of smoking cessation.

The present study supports past research and evidence pointing toward the need to refine current screening eligibility guidelines and eliminate the number of quit-years as an exclusion criterion.^7,15,16^ This need for reform not only applies to the USPSTF but also pertains to the guidelines formulated for the recently implemented national lung cancer screening programs in Germany, Australia, and several other countries.^18,19^ The American Cancer Society (ACS) has made an appeal to the USPSTF to change their current recommendations, advocating against the exclusion of long-term quitters and summarizing a wealth of evidence on the benefits of including this group in screening programs.^16^ A modeling analysis performed for the ACS simulated that eliminating years since quitting from the USPSTF 2021 screening eligibility recommendation increased the number of deaths averted and life-years gained, while the number of overdiagnosed cases remained comparable.^20^ Quit-years were originally used as an eligibility criterion based on the assumption that lung cancer risk continuously decreases after smoking cessation, which has since been disproven.^7^ An additional analysis performed for the ACS demonstrated that while in the first 5 quit-years lung cancer risk tends to decrease, by 10 quit-years, the risk may rebound, potentially resulting in an overall increase in lung cancer risk after 15 years, when the effect of concomitant aging has partially counteracted benefits of prolonged smoking cessation time.^7^

As national lung cancer screening programs expand and validated risk prediction models, such as the Lung Cancer Risk Assessment Tool^21^ and the PLCOm2012 model,^22^ are integrated, the proposed starting ages can direct the timely use of these models for further risk stratification. If former heavy smokers with prolonged quit-times begin annual risk assessments at 50 years of age, their predicted risk would likely initially fall under the screening threshold, giving a misleading indication of low long-term risk. This could reduce motivation for repeated assessments as well as cause unnecessary psychological distress and potential miscommunication regarding future risk and screening eligibility. Guidelines on when to begin risk assessment that are tailored to smoking cessation history could therefore be a useful preceding step before the use of these risk models. Ultimately, our suggested approach of risk-adapted screening ages for former smokers not only offers a practical approach within the current framework, where criteria-based selection serves as the standard, but could also support the emerging use of risk prediction models and future developments in risk-based screening.

In practice, eliminating the years-since-quitting exclusion criterion would expand eligibility to older individuals, necessitating consideration of overall health, longevity, comorbidities, and individual risk before referring to lung cancer screening.^16^ The oldest starting age identified in our study was 67 years, though this was constrained by the age distribution of UKB participants, limiting exploration of risk beyond this age. While the USPSTF sets 80 years as the upper age limit for screening, future research based on decision-analytic modeling could evaluate risk-adapted stopping ages to optimize screening strategies regarding their benefit-harm and cost-effectiveness tradeoffs.^23^

### Strengths and Limitations

In addition to its broad implications, a key strength of this study is that the analyses are based on original individual-level data from the UKB, a large population-based cohort, with large lung cancer case numbers and precise risk estimates.

However, there are several limitations that warrant careful consideration. Although smoking accounts for approximately 80% of all cases globally, there are also other factors that contribute to lung cancer risk, such as environmental and occupational exposures,^24^ which were not included in our analyses. The degree of screening personalization achieved by our proposed approach is therefore limited and could be made more comprehensive in future studies. Specifically, in cohorts where population-level exposure to these risks contributes to a large proportion of cases, it would be useful to incorporate different risk factors for optimized screening starting age calculations. Furthermore, the robustness of our findings is constrained by the data availability in the UKB. Participants older than 70 years were underrepresented in the study cohort. There was also a substantial amount of missing data for smoking variables. There is a risk that replacing missing smoking starting ages with 17 years could, for some individuals, artificially lengthen the smoking duration. However, the effect sizes for quit-time on lung cancer risk were consistent with those derived from the Framingham Heart Study cohort,^25^ suggesting that potential overestimation of the smoking burden is limited. Smoking data may also be vulnerable to recall bias, and the presence of the healthy volunteer effect, selection bias, and participant loss to follow-up has been reported in the UKB.^26^ Furthermore, smoking status could only be updated during follow-up in a small proportion of participants who were repeatedly assessed for smoking data. However, only 2.2% of quitters resumed smoking, indicating that classification of reported former smokers remained correct during follow-up for the vast majority of quitters. Although a higher proportion of self-reported heavy smokers at baseline reported having quit during follow-up, this should not have materially affected the results, as they would still have remained short-term quitters whose risk was similar to that of continuous smokers according to our analysis. Finally, it should be noted that our analysis merely adjusted starting ages based on the USPSTF guidelines recommending 50 years as the earliest eligible age to start screening. We did not, however, assess optimal starting age for current smokers based on benefit-harm or cost-effectiveness ratios. This is the domain of decision-analytic modeling informing health technology assessment. Our derived RPPs could be used to formulate meaningful strategies for such analyses in the future as technology and evidence develops.^23^

## Conclusion

In conclusion, given that LDCT screening does not come without potential harms, a one-size-fits-all approach for population-based lung cancer screening may not be advisable. Importantly, this cohort study supports previous suggestions that former heavy smokers should not be excluded on the mere basis of having quit smoking 15 years prior. On the other hand, for a substantial proportion of former heavy smokers, starting lung cancer screening at an age higher than 50 years might be considered. The expected merits of more refined, but also more complex, risk stratification and starting ages of lung cancer screening for former smokers should be evaluated in further, comprehensive harm-benefit and cost-effectiveness analyses for which our study may provide important input parameters.

## Notes

Supplement 1. **eTable 1.** Missing baseline data in covariates of the study population prior to imputation and restriction to participants with ≥20 pack-years of smoking history **eTable 2.** Baseline characteristics of study participants in sensitivity analysis after imputation of missing smoking data **eTable 3.** Sensitivity analysis: Hazard ratios (HRs), risk postponement periods (RPPs) and risk-adapted starting ages of screening among former smokers, according to years quit smoking

Supplement 2.
Data Sharing Statement
