# Supplementary material for: Risk-Adapted Lung Cancer Screening Starting Ages for Former Smokers
Source: JAMA Netw Open. 2025 Dec 23;8(12):e2551281. doi: 10.1001/jamanetworkopen.2025.51281 (PMC12728645; doi:10.1001/jamanetworkopen.2025.51281)
Supplement: Supplement 1. — eTable 1. Missing baseline data in covariates of the study population prior to imputation and restriction to participants with ≥20 pack-years of smoking history eTable 2. Baseline characteristics of study participants in sensitivity analysis after imputation of missing smoking data eTable 3. Sensitivity analysis: Hazard ratios (HRs), risk postponement periods (RPPs) and risk-adapted starting ages of screening among former smokers, according to years quit smoking [file jamanetwopen-e2551281-s001.pdf]

Supplemental Online Content

Frick C, Hallsson LR, Siebert U, Bhardwaj M, Schöttker B, Brenner H. Risk-adapted lung cancer screening starting ages for former smokers. *JAMA Netw Open*. 2025;8(12):e2551281. doi:10.1001/jamanetworkopen.2025.51281

eTable 1. Missing baseline data in covariates of the study population prior to imputation and restriction to participants with ≥20 pack-years of smoking history

eTable 2. Baseline characteristics of study participants in sensitivity analysis after imputation of missing smoking data

eTable 3. Sensitivity analysis: Hazard ratios (HRs), risk postponement periods (RPPs) and risk-adapted starting ages of screening among former smokers, according to years quit smoking

This supplemental material has been provided by the authors to give readers additional information about their work.

© 2025 Frick C et al. JAMA Network Open.

eTable 1. Missing baseline data in covariates of the study population prior to imputation and restriction to participants with ≥20 pack-years of smoking history

Covariate	Missing No. (%)
Age	0 (0.0)
Sex	0 (0.0)
Quit-years	42,544 (25.4)
Cigarettes per day	54,004 (32.3)
Years smoked	48,581 (29.1)
Pack-years	55,139 (33.0)
Age started smoking	48,224 (28.9)
**All participants**	**166,949 (100)**

Abbreviations: No. = number.
Study population was restricted to participants aged 50 or older (n=117,808), without prevalent lung cancer cases (n=27,116), without unknown smoking status (n=2,146), and excluding never-smokers (n=188,313), as well as individuals with no follow-up or cancer outcome data available (n=24). This table represents data before the final application of eligibility criteria (pack-years ≥ 20), as this restriction was implemented using the imputed pack-years.

© 2025 Frick C et al. JAMA Network Open.

eTable 2. Baseline characteristics of study participants in sensitivity analysis after imputation of missing smoking data

Characteristics	Study participants	
	**Total**<br><br>**No. (%)**	**LC cases**<br><br>**No. (%)**
**All participants**	211,157 (100)	2,741 (100)
**Sex**		
Female	101,302 (48.0)	1,196 (43.6)
Male	109,855 (52.0)	1,545 (56.4)
**Age at baseline, years**		
<40	2 (0.0)	0 (0.0)
40-49	44,206 (21.0)	99 (3.6)
50-59	68,673 (32.5)	650 (23.7)
60-69	97,237 (46.0)	1,969 (71.8)
≥70	1,039 (0.5)	23 (0.8)
**Smoking status at baseline, quit-years** [a]		
Current	50,070 (23.7)	1,291 (47.1)
Former, ≤5 quit-years	28,256 (13.4)	426 (15.5)
Former, 6-10 quit-years	21,091 (10.0)	281 (10.3)
Former, 11-15 quit-years	18,667 (8.8)	180 (6.6)
Former, >15 quit-years	93,073 (44.1)	563 (20.5)
**Years smoked at baseline** [a]		
<15	52,366 (24.8)	145 (5.3)
15-24	51,933 (24.6)	267 (9.7)
25-34	50,762 (24.0)	459 (16.8)
35-44	39,485 (18.7)	977 (35.6)
≥45	16,611 (7.9)	893 (32.6)
**Cigarettes smoked per day**		
≤10	59,989 (28.4)	517 (18.9)
11-20	110,573 (52.4)	1,437 (52.4)
21-30	26,670 (12.6)	482 (17.6)
≥30	13,925 (6.6)	305 (11.1)
**Pack-years, years** [a]		
<20	110,125 (52.1)	555 (20.3)
20-29	41,034 (19.4)	474 (17.3)
30-39	27,786 (13.2)	531 (19.4)
40-49	15,836 (7.5)	503 (18.4)
≥50	16,376 (7.8)	678 (24.7)
**Eligible by USPSTF 2021 criteria** [b]	62,852 (29.8)	1,854 (67.7)

Abbreviations: LC = lung cancer, No. = number.
[a] Years are rounded to the nearest whole number. [b] LC screening eligibility by USPSTF 2021 criteria is as follows: 50-80 years of age, 20 or more pack-years and no more than 15 quit-years.
Characteristics of study participants presented are derived from the first imputed dataset. The number of participants eligible for screening according to the USPSTF criteria differs slightly from Table 1, as it was recalculated following imputation of the study cohort that included individuals younger than 50 years.

© 2025 Frick C et al. JAMA Network Open.

eTable 3. Sensitivity analysis: Hazard ratios (HRs), risk postponement periods (RPPs) and risk-adapted starting ages of screening among former smokers, according to years quit smoking

**Characteristic**	**Person-years [a]**	**LC cases**	**HR [a] (95% CI)**	**RPP, years [a] (95% CI)**	**Risk-adapted starting age [a] (95% CI)**
Age, years			1.11 (1.10, 1.12)	NA	
**Years quit smoking**					
0	467,180	1,279	Ref[b]	NA	50
≤5	74,187	108	0.71 (0.58, 0.88)	3.2 (1.2, 5.2)	53 (51-55)
6-10	190,362	298	0.54 (0.47, 0.61)	6.0 (4.7, 7.2)	56 (55-57)
11-15	216,967	286	0.39 (0.34, 0.45)	9.0 (7.6, 10.3)	59 (58-60)
>15	1,137,813	770	0.20 (0.18, 0.22)	15.3 (14.1, 16.4)	65 (64-66)

Abbreviations: CI = confidence interval, HR = hazard ratio, LC = lung cancer, RPP = risk postponement period
[a] The HRs were rounded to two decimal places, RPPs to one decimal place, and the person-years and risk-adapted starting ages to the nearest whole number.
[b] Participants who currently smoke with ≥20 pack-years of smoking exposure were used as a reference.
The HRs were adjusted for pack-years of smoking history at baseline. Orange shaded cell indicates ineligibility according to USPSTF screening recommendations, blue shaded cells indicate USPSTF eligibility from age 50 onwards.

© 2025 Frick C et al. JAMA Network Open.
