# Supplementary material for: Risk-Adapted Lung Cancer Screening Starting Ages for Former Smokers
Source: JAMA Netw Open. 2025 Dec 23;8(12):e2551281. doi: 10.1001/jamanetworkopen.2025.51281 (PMC12728645; doi:10.1001/jamanetworkopen.2025.51281)
Supplement: Supplement 2. — Data Sharing Statement [file jamanetwopen-e2551281-s002.pdf]

Data Sharing Statement

Frick. Risk-Adapted Lung Cancer Screening Starting Ages for Former Smokers. *JAMA Netw Open*. Published December 23, 2025. doi:10.1001/jamanetworkopen.2025.51281

Data
Data available: No

Additional Information
Explanation for why data not available: Data of the UK Biobank were used which we are not allowed to share. However, researchers can apply for access to these data at the UK Biobank.
